# Surveillance of pyrethroid resistance and detection of voltage-gated sodium channel gene mutations in *Aedes albopictus* populations from Shanghai, China

**DOI:** 10.1186/s13071-025-07035-z

**Published:** 2025-11-24

**Authors:** Kun Qian, Yuqi Dai, Shengfeng Qin, Yang Wang, Xiangkun Meng, Jianjun Wang, Hongxia Liu

**Affiliations:** 1 College of Plant Protection, Yangzhou University, Yangzhou, 225009 China; 2 College of Plant Protection, Jilin Agricultural University, Changchun, 130118 China; 3 Department of Infectious Disease Control, Shanghai Municipal Center for Disease Control and Prevention, Shanghai, 200336 China

**Keywords:** *Aedes albopictus*, Pyrethroid resistance, Sodium channel gene, VGSC mutation detection, LAMP assay

## Abstract

**Background:**

As a global hub for trade and tourism, Shanghai faces escalating risks of dengue fever due to imported and local transmission. *Aedes albopictus*, the primary dengue vector in the region, is predominantly controlled using pyrethroid insecticides. However, widespread resistance threatens their efficacy.

**Methods:**

Resistance levels of *Ae. albopictus* to four pyrethroids (deltamethrin, permethrin, lambda-cyhalothrin, beta-cypermethrin) in 12 Shanghai districts were assessed via adult mosquito contact tube bioassays, with resistance categorized by mortality rates at diagnostic doses. Voltage-gated sodium channel (VGSC) gene mutations in 255 adult mosquitoes were identified through sequencing. A loop-mediated isothermal amplification (LAMP) assay was developed to rapidly detect the F1534S mutation in the VGSC gene.

**Results:**

The Baoshan district population exhibited resistance to cyhalothrin (mortality: 80.3%), while populations in all other districts displayed confirmed resistance (mortality < 80%) to all four pyrethroids. VGSC mutation analysis revealed high frequencies of F1534S (84.71%), moderate V1016G (21.96%), and rare I1532T (1.57%). An optimized LAMP assay for F1534S detection was established with the following parameters: 0.5 U/μl Bst DNA polymerase, 0.8 mM deoxynucleoside triphosphates (dNTPs), 3.00 mM Mg^2+^, 1.00 mM betaine, 2.00 μM forward/backward inner primers (FIP/BIP), 0.40 μM F3/B3 primers, 0.8 mM hydroxyl naphthol blue (HNB), and 60-min incubation at 63 °C. The assay demonstrated 100% concordance with sequencing results and high sensitivity.

**Conclusions:**

Pyrethroid resistance is pervasive in Shanghai’s *Ae. albopictus* populations, driven by high-frequency VGSC mutations. The LAMP assay provides a rapid field-deployable tool for resistance monitoring, guiding targeted vector control strategies.

**Graphical Abstract:**

**Figure:**
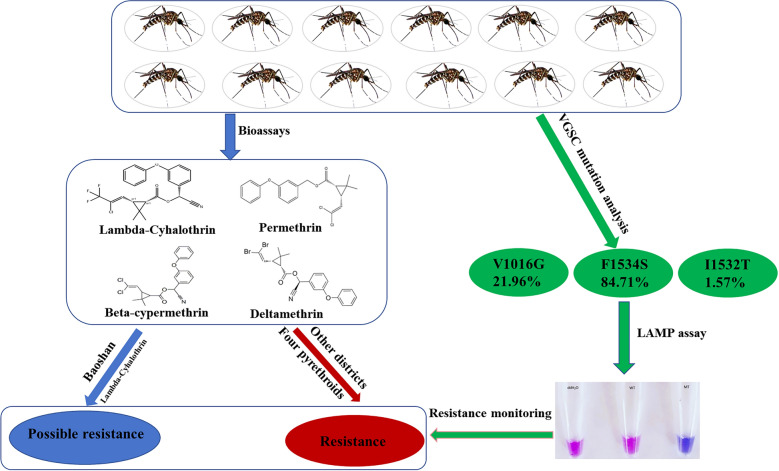

## Background

*Aedes albopictus* (Asian tiger mosquito), a small to medium-sized species within the family Culicidae, exhibits remarkable ecological plasticity, enabling rapid adaptation to diverse habitats ranging from natural ecosystems to urban environments [1]. Native to Southeast Asia and the Western Pacific Islands, this invasive mosquito has expanded its global distribution to over 70 countries across North America, Europe, Africa, and beyond, driven by intensified international trade and human mobility [2]. Recognized as a primary vector for dengue, chikungunya, and Zika viruses, *Ae. albopictus* poses a significant threat to global public health. Dengue alone affects approximately 390 million people annually, underscoring the critical reliance on insecticide-based vector control strategies endorsed by the World Health Organization (WHO) [3].

Pyrethroid insecticides, prized for their efficacy and low mammalian toxicity, remain the cornerstone of chemical interventions against *Ae. albopictus*. However, their prolonged and widespread use has accelerated the evolution of resistance, primarily mediated by target-site insensitivity (e.g., knockdown resistance) and metabolic detoxification [4]. Knockdown resistance (*kdr*) arises from mutations in the voltage-gated sodium channel (VGSC) gene, which decreases mosquito sensitivity to pyrethroids by altering the insecticide binding site [5]. Notably, mutations such as F1534S and V1016G in the VGSC gene have been strongly associated with high-level pyrethroid resistance in *Ae. albopictus* populations across Asia [6, 7].

Shanghai, a global metropolis characterized by dense populations and frequent international exchanges, faces escalating risks of imported dengue outbreaks. While pyrethroids are central to local *Ae. albopictus* control, recent surveillance indicates rising resistance levels, threatening the sustainability of current strategies [8]. Despite this urgency, systematic data on the geographical distribution of resistance, VGSC mutation profiles, and their correlation with phenotypic resistance across Shanghai’s districts remain scarce. To address this gap, this study investigates pyrethroid resistance levels in *Ae. albopictus* populations from 12 administrative districts of Shanghai, analyzes the frequency of VGSC resistance-associated mutations, and develops a loop-mediated isothermal amplification (LAMP) assay for rapid detection of the *kdr* mutation. These findings aim to elucidate resistance mechanisms, inform targeted insecticide management, and enhance dengue prevention frameworks in high-risk urban settings.

## Methods

### Mosquito collection and rearing

**Fig. 1:**
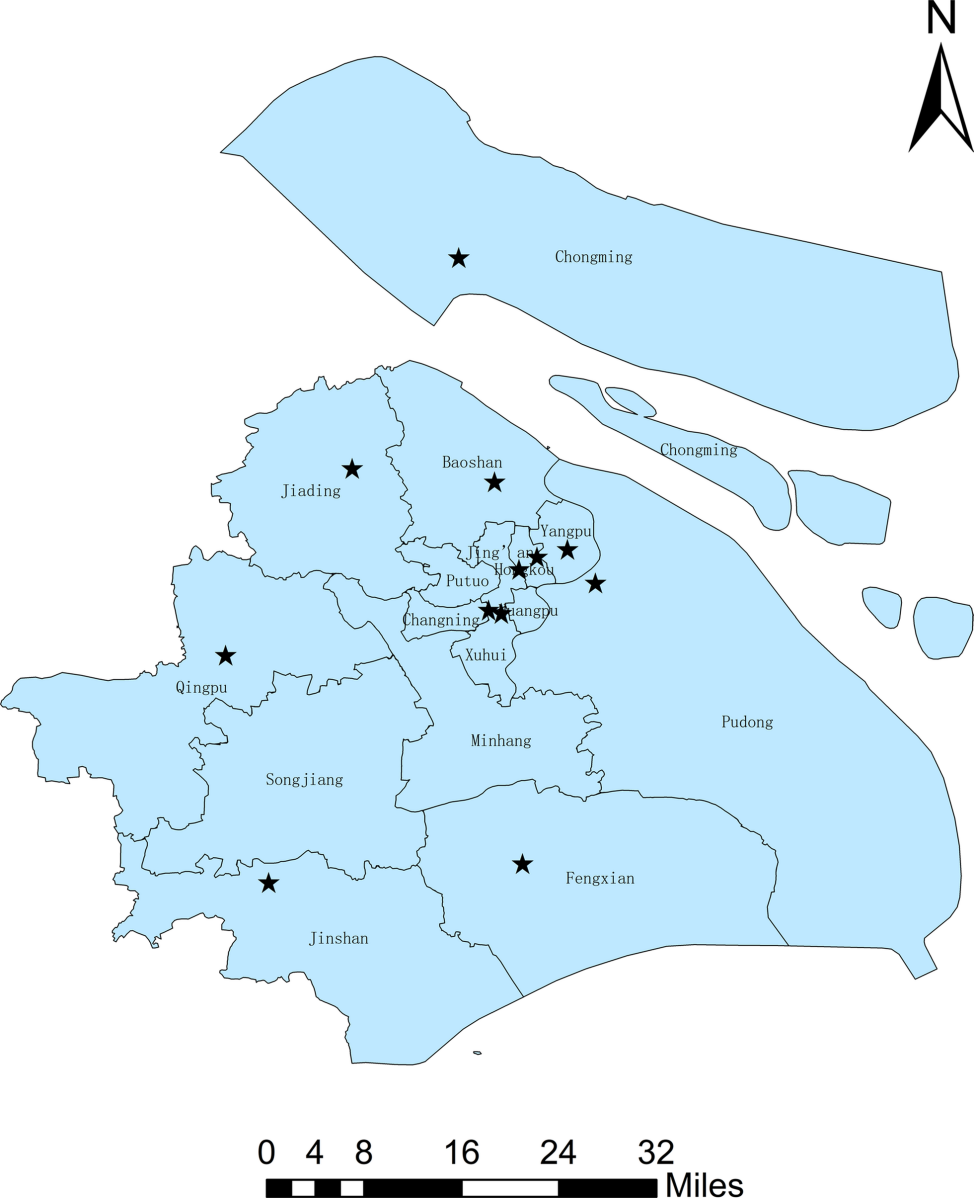
Map of collection locations of *Ae. albopictus* in Shanghai

*Aedes albopictus* larvae were collected from 12 distinct geographical locations in Shanghai (Table 1; Fig. 1) and subsequently reared under controlled laboratory conditions. Adult female mosquitoes (first filial [F1] generation) were maintained for 3–5 days post-emergence prior to insecticide susceptibility testing. Following bioassay, specimens were immediately flash-frozen in liquid nitrogen and subsequently stored in a −80 °C ultralow-temperature freezer for long-term preservation of biomolecular integrity.

**Table 1:** Brief information of the sampling location in Shanghai

Sampling location	Code	Species	Longitude	Latitude	Date
Bao Shan	BS	*Aedes albopictus*	121.44000	31.37000	July–August 2021
Chong Ming	CM	*Ae. albopictus*	121.39752	31.63626	July–August 2021
Chang Ning	CN	*Ae. albopictus*	121.43293	31.21771	July–August 2021
Feng Xian	FX	*Ae. albopictus*	121.47342	30.91690	July–August 2021
Hong Kou	HK	*Ae. albopictus*	121.29260	31.16510	July–August 2021
Jing An	JA	*Ae. albopictus*	121.47922	31.26607	July–August 2021
Jia Ding	JD	*Ae. albopictus*	121.27078	31.38593	July–August 2021
Jin Shan	JS	*Ae. albopictus*	121.17171	30.89455	July–August 2021
Pu Dong	PD	*Ae. albopictus*	121.56000	31.25000	July–August 2021
Qing Pu	QP	*Ae. albopictus*	121.12042	31.15139	July–August 2021
Xu Hui	XH	*Ae. albopictus*	121.44818	31.21388	July–August 2021
Yang Pu	YP	*Ae. albopictus*	121.52674	31.28981	July–August 2021

### Insecticide susceptibility testing

Insecticide susceptibility testing was conducted following the standardized contact tube method specified in the Guidelines for Bioassay of Mosquito Resistance Detection Methods (GB/T 26347-2010, https://www.nhc.gov.cn/wjw/s9498/201106/51938.shtml). Diagnostic doses were determined as 0.07% lambda-cyhalothrin, 0.08% beta-cypermethrin, 0.4% permethrin, and 0.03% deltamethrin (provided by the Chinese Center for Disease Control and Prevention, China CDC, corresponding to twice the 99% lethal concentration [LC_99_] of the reference susceptible strain).

Non-blood-fed female mosquitoes (3–5 days post-emergence) were randomly selected and exposed to insecticide-impregnated filter papers in WHO-approved contact tubes (245 cm^3^ volume, *n* = 30 per replicate). After 1-h exposure under controlled conditions (25 ± 1 °C, 70 ± 5% relative humidity), mosquitoes were gently transferred to recovery containers with 10% glucose solution. Mortality was assessed after a 24-h holding period. Each treatment included triplicate experimental groups and parallel blank controls (untreated filter paper). Mortality in blank controls was consistently < 5%, confirming valid bioassay conditions. Resistance classification criteria were as follows: mortality at diagnostic dose; susceptible population, 98–100%; potential resistance, 80–97% mortality; confirmed resistance, < 80% mortality.

### Amplification and sequence analysis of VGSC fragments

Genomic DNA was isolated from 255 adult *Ae. albopictus* mosquitoes (F1 generation, morphologically identified and unexposed to insecticides) using the MiniBEST Universal Genomic DNA Extraction Kit version 5.0 (Takara) per the manufacturer’s instructions. The target fragments spanning transmembrane domains II and III of the VGSC gene were amplified using genomic DNA as the template, with primers synthesized according to the method described by Kasai et al. [9, 10]. The amplification/sequencing primer sequences are detailed in Table 2. Domain II partial sequences (500 base pairs [bp] covering codon 1016) were amplified using primers V2F/V2R, while primers V3F/V3R targeted domain III fragments (700 bp covering codons 1532/1534). The polymerase chain reaction (PCR) reaction mixture contained 25 μl of 2× FastTaq Premix (with dye), 1 μl each of 10 nmol forward and reverse primers, and 2 μl of DNA template, and was adjusted to a final volume of 50 μl with double-distilled (dd)H_2_O. Thermal cycling conditions were as follows: 95 °C for 5 min (initial denaturation); 35 cycles of 95 °C for 30 s (denaturation), 52 °C for 30 s (annealing), and 72 °C for 30 s (extension); followed by a final extension at 72 °C for 10 min and a holding step at 4 °C.

**Table 2:** Brief information about amplification and sequencing primer in this study

Primer name	Sequence (5′–3′)
V2F (Forward amplification primer)	5′-GACAATGTGGATGGCTTCCC-3′
V2R (Reverse amplification primer)	5′-GCAATCTGGCTTGTTAACTTG-3′
V2R-2 (Reverse sequencing primer)	5′-TTCACGAACTTGAGCGCGTTG-3′
V3F (Forward amplification primer)	5′-GAGAACTCGCCGATGAACTT-3′
V3R (Reverse amplification primer)	5′-GACGACGAAATCGAACAGGT-3′
V3R-3 (Reverse sequencing primer)	5′-TAGCTTTCAGCGGCTTCTTC-3′

The stable portion of the sequencing results was extracted. Sequence alignment and map analysis were then performed using Vector NTI and SnapGene software. The sequences obtained from the V2 and V3 sequencing systems were compared with reference sequences from GenBank (accession numbers KC152045 and KC152046, respectively). Finally, Excel software was used to statistically analyze and calculate the mutant genotypes and allele frequencies of *Ae. albopictus* in different regions.

### LAMP primer design

Target-specific LAMP primers were designed using Primer Explorer V5 (Eiken Chemical Co., Ltd., Japan; https://primerexplorer.jp/e/) to detect the mutation in the VGSC gene of *Ae. albopictus*, adhering to established thermodynamic parameters for LAMP assays. The design criteria included GC content of 40–65% to ensure optimal primer–template binding stability, with amplicon architectural constraints specifying an F2–B2 spacer length of 120–180 bp, an F2–F1 interval of 40–60 bp, and an F3–F2 gap of 0–50 bp in the 5′ → 3′ direction. Secondary structure parameters required an F2–B1 distance of 40–60 bp and a B3–B2 separation of 0–60 bp in the 3′ → 5′ orientation. Primer specificity was confirmed in silico using the nucleotide Basic Local Alignment Search Tool (BLASTn) (National Center for Biotechnology Information [NCBI]) against the *Ae. albopictus* genome reference in VectorBase.

### LAMP reaction system

Components (Table 3) were added sequentially to a 200-μl tube to prepare the 15-μl reaction mixture [11], then incubated at 63 °C for 60 min in a metal bath.

**Table 3:** LAMP reaction system

Component	Volume (μl)
Bst DNA polymerase (8U/µl)	0.6
Buffer (10× ThermoPol reaction buffer)	1.5
MgSO_4_ (100 mM)	0.6
dNTPs (10 mM)	1.5
FIP (10 µM)	2.4
BIP (10 µM)	2.4
F3 (10 µM)	0.6
B3 (10 µM)	0.6
Betaine (5 mM)	1.5
HNB (2.4 mM)	1.0
DNA template	0.6
ddH_2_O	1.7

*BsT* *Bacillus stearothermophilus*, *HNB* hydroxyl naphthol blue, *ddH*_*2*_*O* double-distilled water

### LAMP system optimization

To reduce detection costs, optimization was performed on the components of the LAMP reaction system. The concentrations of various components were adjusted as follows: Bst DNA polymerase (0.1, 0.2, 0.3, 0.4, and 0.5 U/µl); deoxynucleoside triphosphates (dNTPs) (0.2, 0.4, 0.6, 0.8, and 1.0 mM); Mg^2+^ (1.0, 2.0, 3.0, 4.0, and 5.0 mM); betaine (0.2, 0.4, 0.6, 0.8, and 1.0 M); forward inner primer (FIP)/backward inner primer (BIP) (0.4, 0.8, 1.2, 1.6, and 2.0 µM); F3/B3 (0.1, 0.2, 0.3, 0.4, and 0.5 µM); and hydroxyl naphthol blue (HNB) (0.2, 0.4, 0.6, 0.8, and 1.0 mM). The optimal concentrations were ultimately determined through HNB colorimetric analysis. To obtain optimal reaction parameters, optimization of LAMP reaction temperature and duration was conducted based on the established optimal concentrations of system components. Isothermal amplification was first performed at different temperatures (61 °C, 62 °C, 63 °C, 64 °C, and 65 °C) for 60 min to determine the optimal temperature. Subsequently, time course experiments were carried out at the optimized temperature using multiple time points (30, 40, 50, 60, and 70 min). The optimal temperature–time combination was ultimately determined through HNB colorimetric analysis.

### Statistical analyses

All data in this study are presented as the mean ± standard error of the mean (SEM) (GraphPad Software, Inc., San Diego, CA, U.S.A.). Statistical analysis was performed using Student’s *t*-test (SPSS version 10.0, SPSS Inc., Chicago, IL, USA).

## Results

### Resistance of *Ae. albopictus* to pyrethroid pesticides

The resistance profiles of adult *Ae. albopictus* mosquitoes to four pyrethroid insecticides (lambda-cyhalothrin, beta-cypermethrin, permethrin, and deltamethrin) across 12 geographical areas in Shanghai are summarized in Table 4, with mortality rates (mean ± standard deviation) and resistance categories (S: susceptible, M: possibly resistant, R: resistant) reported. Resistance categories followed GB/T 26347–2010 (S: 98–100%, M: 80–97%, R: < 80%). For lambda-cyhalothrin, mortality ranged from 6.32% (CM) to 80.3% (BS), with BS classified as “possibly resistant” (M) and all other areas categorized as resistant (R). Beta-cypermethrin exhibited mortality rates ranging from 14.96% (PD) to 77.6% (BS), with all areas designated as resistant (R). Permethrin demonstrated mortality extremes of 6.33% (XH) and 65.98% (FX), while deltamethrin showed the widest variability, ranging from 2.78% (YP) to 73.1% (BS), although all regions uniformly maintained resistant (R) status. Resistance (R) predominated across all insecticides and regions except for lambda-cyhalothrin in BS (M). These results underscore the pervasive pyrethroid resistance in Shanghai’s *Ae. albopictus* populations, with marked geographical variability in insecticide-specific mortality outcomes.

**Table 4:** Test results of resistance of adult *Ae. albopictus* mosquitoes to four pyrethroid insecticides in the Shanghai area

	Lambda-cyhalothrin			Beta-cypermethrin		Permethrin		Deltamethrin	
Area	Mortality	Resistance	Mortality		Resistance	Mortality	Resistance	Mortality	Resistance
	± SD (%)		± SD (%)			± SD (%)		± SD (%)	
BS	80.3 ± 4.98a	M	77.6 ± 7.28a		R	50.6 ± 5.05a	R	73.1 ± 0.73a	R
CM	6.32 ± 2.18d	R	62.22 ± 1.00a		R	15.56 ± 4.67c	R	10.42 ± 1.88c	R
CN	56.14 ± 8.93b	R	52.13 ± 14.52a		R	27.71 ± 2.61b	R	45.10 ± 8.48b	R
FX	14.00 ± 4.59c	R	63.00 ± 10.29a		R	65.98 ± 5.32a	R	42.72 ± 10.76b	R
HK	51.84 ± 8.29a	R	65.32 ± 13.15a		R	18.58 ± 16.60b	R	53.79 ± 8.52a	R
JA	42.17 ± 5.12b	R	55.29 ± 3.60a		R	40.24 ± 6.17b	R	41.38 ± 2.24b	R
JD	67.90 ± 3.86a	R	33.80 ± 3.50b		R	44.00 ± 4.88b	R	28.00 ± 4.42b	R
JS	28.26 ± 8.20c	R	27.66 ± 7.19b		R	17.20 ± 3.09b	R	27.03 ± 10.27c	R
PD	16.20 ± 3.20c	R	14.96 ± 6.57b		R	15.12 ± 7.39c	R	16.33 ± 8.72c	R
QP	65.64 ± 4.86a	R	17.14 ± 5.12b		R	21.11 ± 8.42b	R	43.95 ± 12.06b	R
XH	25.30 ± 14.74c	R	62.07 ± 11.25a		R	6.33 ± 4.59c	R	37.50 ± 5.92b	R
YP	77.91 ± 11.98a	R	39.29 ± 20.26b		R	39.56 ± 13.63b	R	2.78 ± 2.79c	R

S = susceptible population; M = possibly resistant population; R = resistant population. All data are expressed as the mean ± standard deviation (SD). Different letters (a, b, c, d) indicate significant differences (*p* < 0.05) in mortality among districts for the same insecticide (one-way analysis of variance (ANOVA)]

### Distribution and frequency of VGSC genotypes

**Fig. 2:**
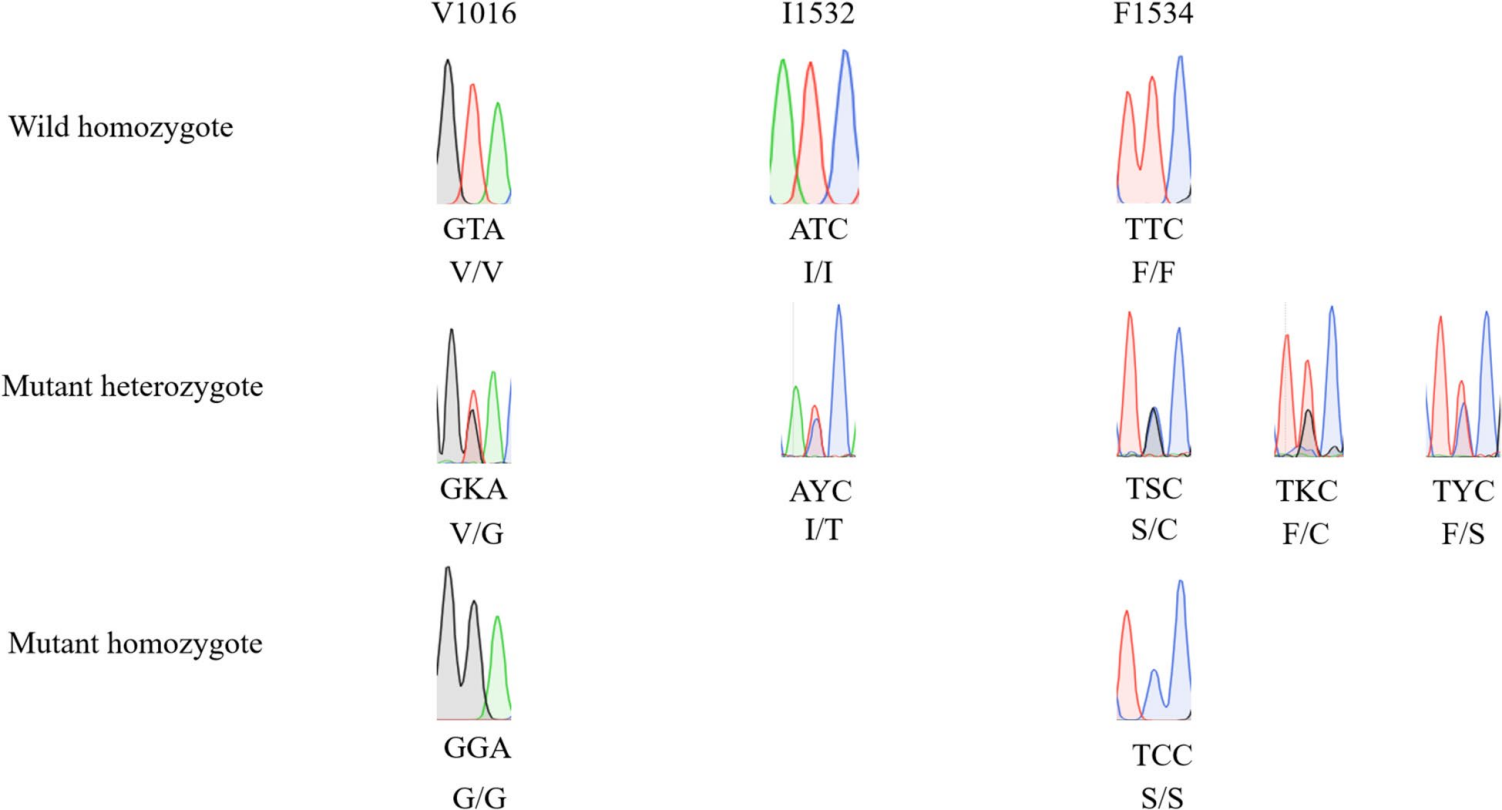
Sequencing chromatograms of voltage-gated sodium channel (VGSC) gene loci 1016, 1532, and 1534 in *Ae. albopictus*. Wild-type homozygotes include V/V (codon GTA), I/I (codon ATC), and F/F (codon TTC). Mutant heterozygotes comprise V/G (codon GKA, K = T/G), I/T (codon AYC, Y = C/T), F/S (codon TYC, Y = T/C), S/C (codon TSC, S = G/C), and F/C (codon TKC, K = G/T). Mutant homozygotes are G/G (codon GGA) and S/S (codon TCC). Codon abbreviations (e.g., GTA, ATC) represent nucleotide triplets; degenerate bases (K, S, Y) are defined as K = T/G, S = G/C, Y = C/T. Genotypes denote amino acid variants (e.g., V/V: valine homozygote)

The genotype frequencies at loci V1016, I1532, and F1534, critical markers associated with insecticide resistance in *Ae. albopictus*, were comprehensively characterized using sequencing across 12 geographically distinct districts of Shanghai (Table 5; Fig. 2). Small sample sizes in BS (*n* = 5) and CN (*n* = 4) reflect field collection constraints; trends should be interpreted cautiously. The homozygous V/V genotype at locus V1016 was dominant overall (78.04%), with frequencies exceeding 75% in 10 out of 12 districts, including BS, CN, FX, and HK. Exceptions included districts CM and JD, where the heterozygous V/G genotype reached 36.36% and 47.37%, respectively. The homozygous G/G genotype was rare (1.57% total) and was observed exclusively in JA (20.00%). At locus I1532, the homozygous I/I genotype was nearly fixed (98.43% total), with minor deviations in CN (75.00% I/I and 25.00% heterozygous I/T) and JS (93.75% I/I and 6.25% I/T). No T/T homozygotes were detected in any district. For locus F1534, the S/S genotype dominated overall (56.47%), with particularly high frequencies in BS (80.00%), FX (83.33%), and XH (91.67%). District CM exhibited a distinct profile, characterized by high frequencies of F/F (40.91%) and F/S (59.09%). Rare variants F/C and S/C were detected in JA (5.00% F/C) and HK (10.71% S/C). Collectively, loci V1016 and I1532 displayed limited genetic variation across districts, while F1534 exhibited substantial diversity, suggesting potential regional differences in evolutionary pressures or genetic dynamics.

**Table 5:** Genotypes and frequencies (%) of loci 1016, 1532, and 1534 of *Ae. albopictus* population in 12 districts of Shanghai

Number	Area	*N*	V1016			I1532			F1534				
			V/V	V/G	G/G	I/I	I/T	T/T	F/F	F/S	S/S	F/C	S/C
1	BS	5	100.00	0.00	0.00	100.00	0.00	0.00	20.00	0.00	80.00	0.00	0.00
2	CM	22	63.64	36.36	0.00	100.00	0.00	0.00	40.91	59.09	0.00	0.00	0.00
3	CN	4	100.00	0.00	0.00	75.00	25.00	0.00	25.00	0.00	75.00	0.00	0.00
4	FX	24	91.67	8.33	0.00	100.00	0.00	0.00	0.00	16.67	83.33	0.00	0.00
5	HK	28	100.00	0.00	0.00	100.00	0.00	0.00	14.29	17.86	57.14	0.00	10.71
6	JA	20	30.00	50.00	20.00	95.00	5.00	0.00	25.00	25.00	45.00	5.00	0.00
7	JD	19	52.63	47.37	0.00	100.00	0.00	0.00	15.79	68.42	15.79	0.00	0.00
8	JS	32	81.25	18.75	0.00	93.75	6.25	0.00	6.25	25.00	65.63	3.13	0.00
9	PD	25	80.00	20.00	0.00	100.00	0.00	0.00	12.00	24.00	64.00	0.00	0.00
10	QP	16	87.25	12.50	0.00	100.00	0.00	0.00	18.75	37.50	43.75	0.00	0.00
11	XH	24	75.00	25.00	0.00	100.00	0.00	0.00	0.00	8.33	91.67	0.00	0.00
12	YP	36	88.89	11.11	0.00	100.00	0.00	0.00	8.33	27.78	63.89	0.00	0.00
Total		255	78.04	20.39	1.57	98.43	1.57	0.00	13.33	28.24	56.47	0.78	1.18

Table 6 shows the frequency distribution of 14 combinatorial *kdr* mutant genotypes (G1–G14) involving V1016, I1532, and F1534 substitutions across 12 geographical areas (total *N* = 255). Genotype G5 (V/V + I/I + S/S) is overwhelmingly dominant overall (52.55%) and in most individual areas, while G4 (V/V + I/I + F/S) is the second most prevalent (18.04%). Wild-type G1 (V/V + I/I + F/F) occurs at 7.45% overall. Significant regional variation exists, with CM showing high G1 and G4 frequencies (both 31.82%), JD exhibiting high G4 (42.11%) and G8 (26.32%), and JA/JS displaying the greatest genotypic diversity. Most other genotypes are rare (≤ 3.92%) or absent in many areas.

**Table 6:** Combinatorial mutant genotypes and frequencies (%) distribution of V1016 + I1532 + F1534 loci in the VGSC gene of *Ae. albopictus*

Number	Area	*N*	G1	G2	G3	G4	G5	G6	G7	G8	G9	G10	G11	G12	G13	G14
1	BS	5	20.00	0.00	0.00	0.00	80.00	0.00	0.00	0.00	0.00	0.00	0.00	0.00	0.00	0.00
2	CM	22	31.82	0.00	0.00	31.82	0.00	0.00	0.00	27.27	9.09	0.00	0.00	0.00	0.00	0.00
3	CN	4	0.00	0.00	0.00	0.00	75.00	25.00	0.00	0.00	0.00	0.00	0.00	0.00	0.00	0.00
4	FX	24	0.00	0.00	0.00	12.50	83.33	0.00	0.00	4.17	0.00	0.00	0.00	0.00	0.00	0.00
5	HK	28	14.29	0.00	10.71	17.86	57.14	0.00	0.00	0.00	0.00	0.00	0.00	0.00	0.00	0.00
6	JA	20	5.00	0.00	0.00	10.00	30.00	0.00	0.00	10.00	5.00	15.00	5.00	5.00	10.00	5.00
7	JD	19	0.00	0.00	0.00	42.11	10.53	0.00	0.00	26.32	15.79	5.26	0.00	0.00	0.00	0.00
8	JS	32	0.00	3.13	0.00	12.50	62.50	0.00	3.12	9.38	3.13	3.13	3.13	0.00	0.00	0.00
9	PD	25	8.00	0.00	0.00	12.00	60.00	0.00	0.00	12.00	4.00	4.00	0.00	0.00	0.00	0.00
10	QP	16	18.75	0.00	0.00	25.00	43.75	0.00	0.00	12.50	0.00	0.00	0.00	0.00	0.00	0.00
11	XH	24	0.00	0.00	0.00	8.33	79.17	0.00	0.00	0.00	0.00	12.5	0.00	0.00	0.00	0.00
12	YP	36	2.78	0.00	0.00	22.22	61.11	0.00	0.00	2.78	5.56	2.78	0.00	2.78	0.00	0.00
Total		255	7.45	0.39	1.18	18.04	52.55	0.39	0.39	9.02	3.92	3.92	0.78	0.78	0.78	0.39

G1: V/V + I/I + F/F; G2: V/V + I/I + F/C; G3: V/V + I/I + S/C; G4: V/V + I/I + F/S; G5: V/V + I/I + S/S; G6: V/V + I/T + F/F; G7: V/V + I/T + F/S; G8: V/G + I/I + F/S; G9: V/G + I/I + F/F; G10: V/G + I/I + S/S;

G11: V/G + I/T + F/F; G12: G/G + I/I + F/S; G13: G/G + I/I + F/F; G14: G/G + I/I + F/C

### LAMP primer design and screening

**Fig. 3:**
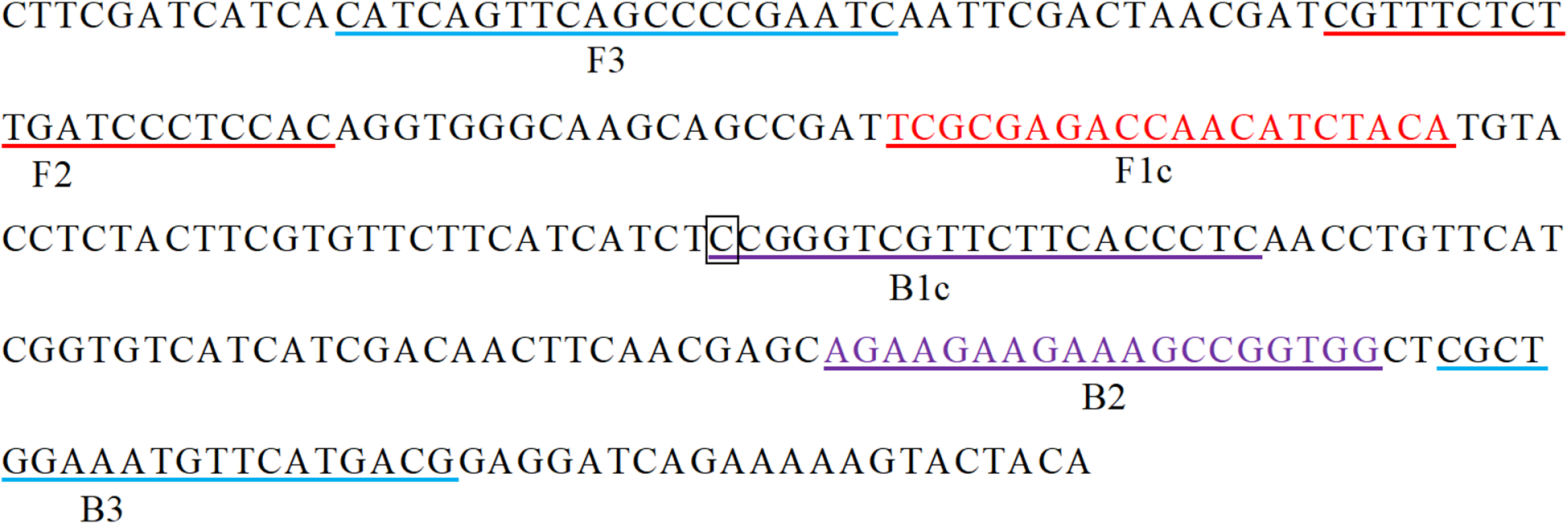
LAMP primer design drawing. Primers marked in red font are the reverse primer F1c, and primers marked in purple font are the reverse primer B2. FIP consists of the reverse primer F1c and the forward primer F2, while BIP is composed of the forward primer B1c and the reverse primer B2. The bases enclosed in black square boxes represent mutated bases

Based on the mutated VGSC gene sequence (GenBank no. Kc.152046) of *Ae. albopictus* (position 1534), LAMP primers were designed (Fig. 3) and screened for specificity/sensitivity. One optimal primer set was identified from 20 candidates (Table 7).

**Table 7:** List of LAMP primers

Primer name	Sequence (5’–3′)
F3	CATCAGTTCAGCCCCGAATC
B3	CGCTCATGAACATTTCCAGCG
FIP	TGTAGATGTTGGTCTCGCGA-CGTTTCTCTTGATCCCTCCAC
BIP	CCGGGTCGTTCTTCACCCTC-CCACCGGCTTTCTTCTTCTG

### Rapid detection of F1534S mutation using LAMP technology

**Fig. 4:**
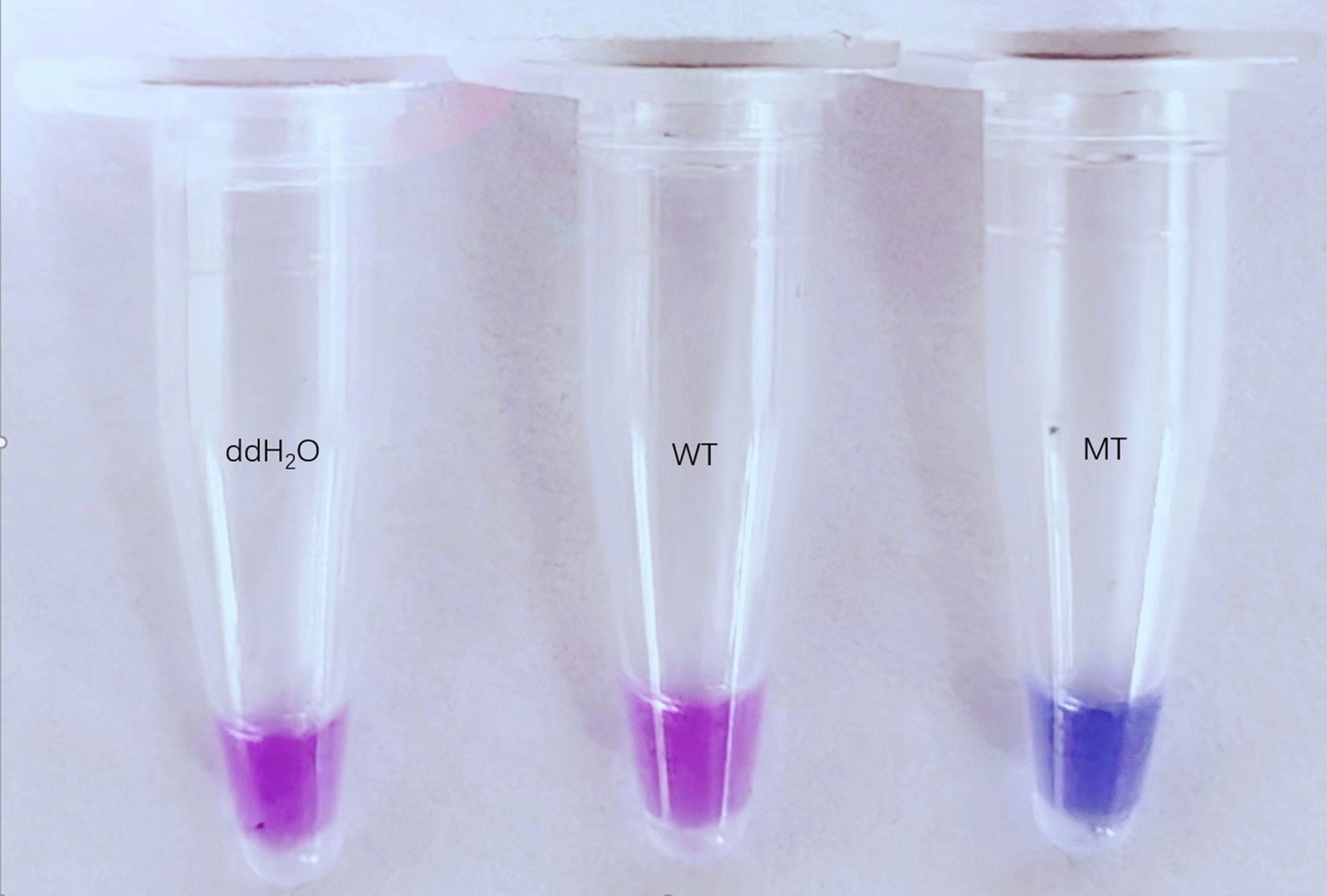
HNB colorimetric results. ddH_2_O (double-distilled water) and WT (wild type) represent the negative control and susceptible control, respectively, while MT (mutant type) serves as the resistant control

Twenty samples were randomly selected from 12 successfully amplified populations in Shanghai for allele-specific (AS)-LAMP analysis. As illustrated in Fig. 4, the HNB indicator shifted from purple to sky blue upon successful amplification using specific primers. Samples with ≥ 1 S allele (F/S or S/S) turned blue; wild-type (F/F) remained purple. In contrast, reactions containing ddH_2_O or F/F genotype DNA templates retained the original purple coloration. The assay encompassed nine S/S genotype mutants, six F/S genotype mutants, and five F/F wild genotype. Results demonstrated that all 15 samples harboring TCC/S-type alleles exhibited the characteristic blue color transition, while F/F genotype samples and ddH_2_O controls remained unchanged, with persistent purple HNB visualization. These detection outcomes showed complete concordance with sequencing validation data, confirming the assay's specificity and reliability in allele differentiation.

### The optimized composition of the LAMP reaction system

**Fig. 5:**
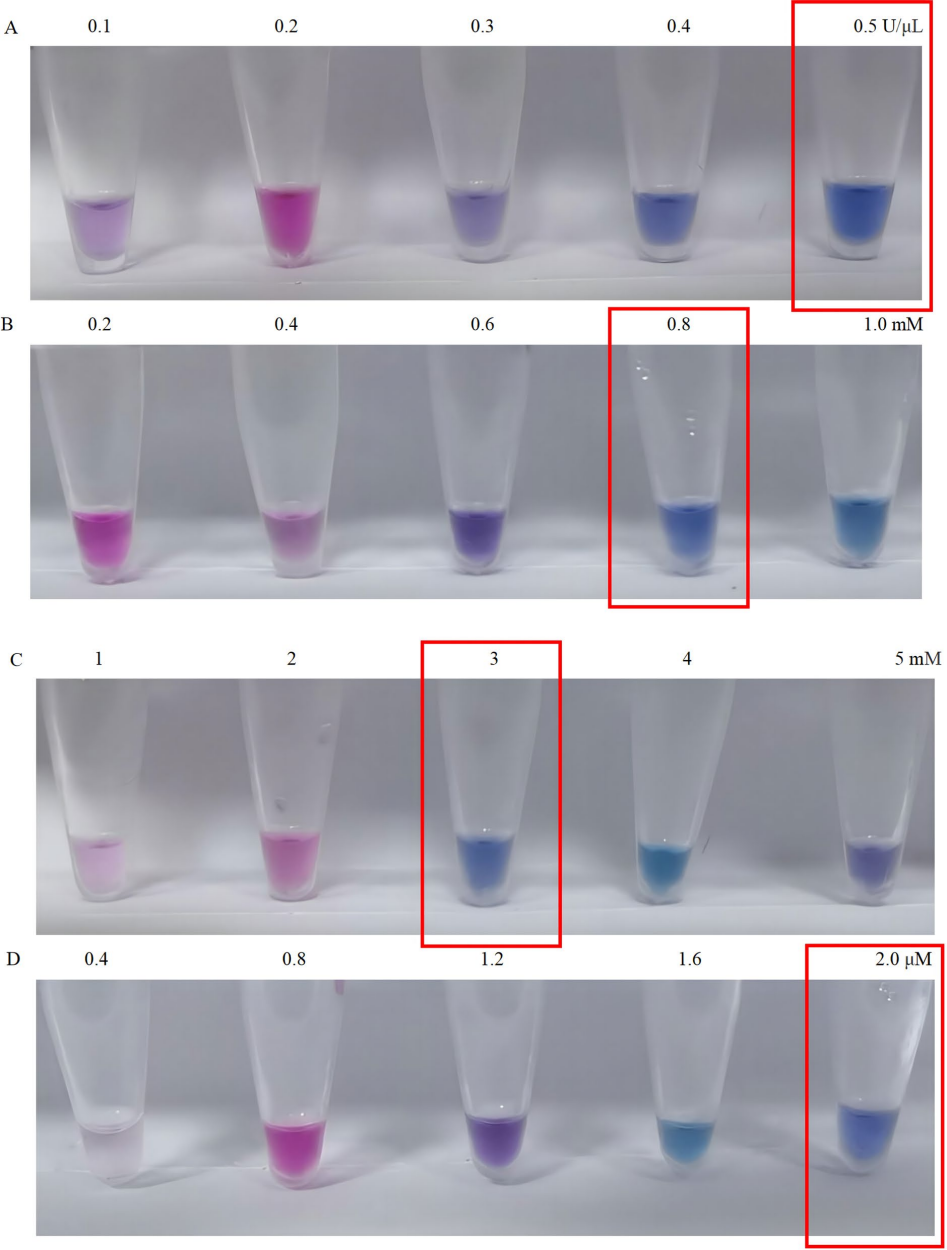
Optimization results of Bst DNA polymerase, dNTP, Mg^2+^, and FIP/BIP concentrations in the LAMP reaction system. Panels **A**, **B**, **C**, and **D** represent the reaction performance under different concentration gradients of Bst DNA polymerase, dNTP, Mg^2+^, and FIP/BIP, respectively. The optimal reaction concentrations are indicated within the red boxes

**Fig. 6:**
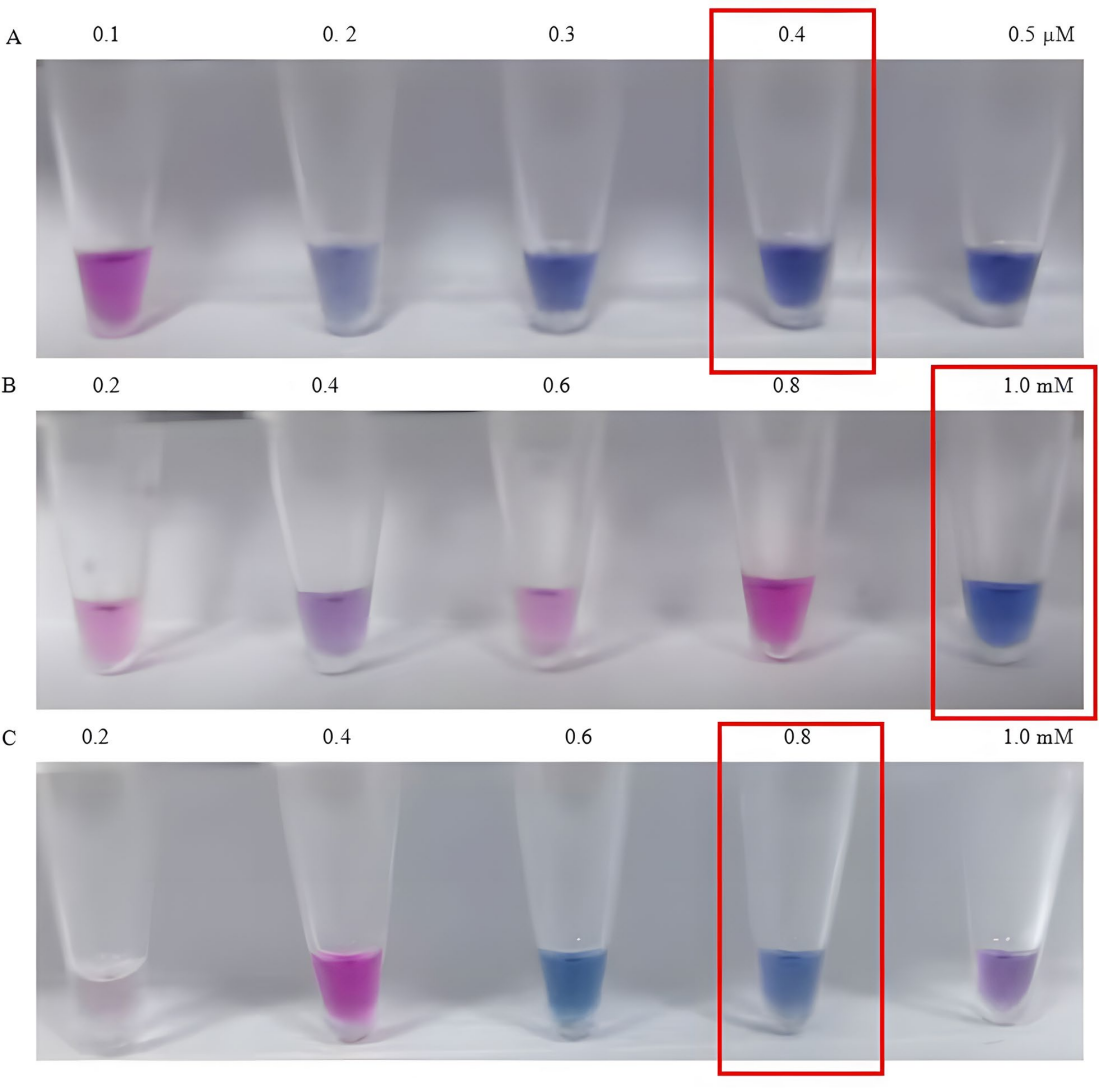
Optimization results of F3/B3 primers, betaine, and HNB in the LAMP reaction system. Panels **A**, **B**, and **C** represent the reaction performance under different concentration gradients of F3/B3 primers, betaine, and HNB, respectively. The optimal reaction concentrations are indicated within the red boxes

**Fig. 7:**
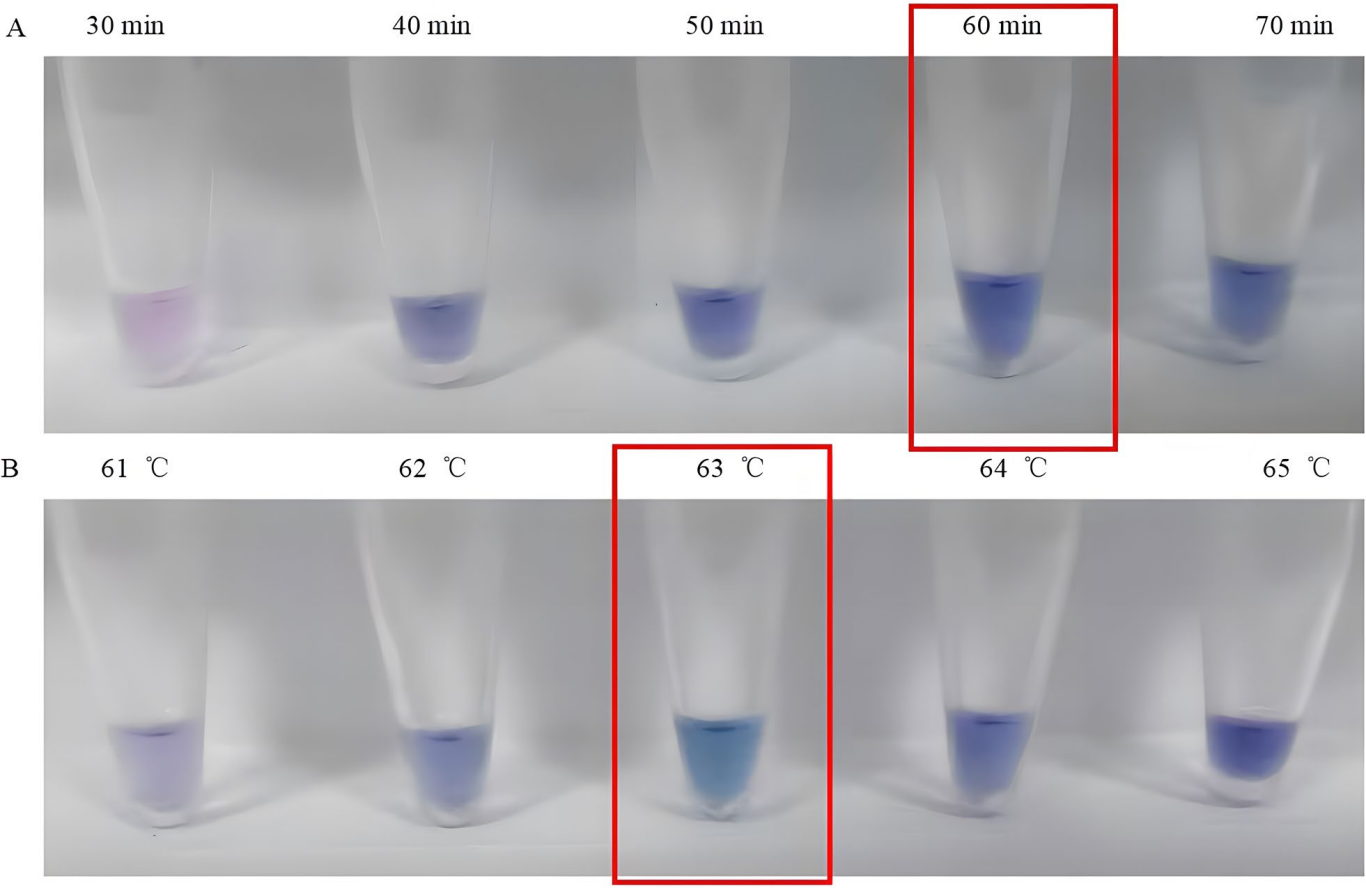
Optimization of reaction temperature and time for LAMP. Panels **A** and **B** represent the LAMP reaction performance under different reaction times and temperatures, respectively. The optimal reaction time and temperature are indicated within the red boxes

**Fig. 8:**
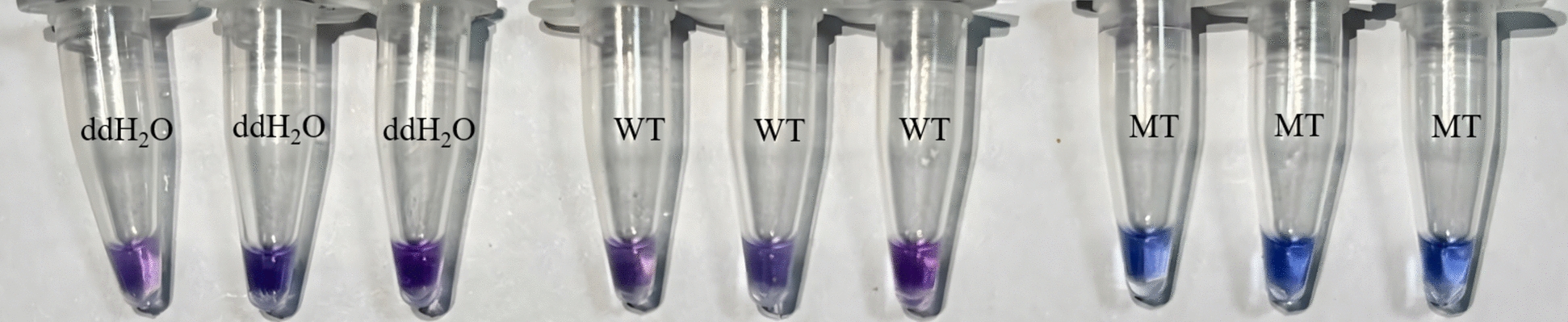
HNB colorimetric results (optimized reaction). ddH_2_O (double-distilled water) and WT (wild type) represent the negative control and susceptible control, respectively, while MT (mutant type) serves as the resistant control

As shown in Figs. 5 and 6, the optimal reaction concentrations of each component were Bst DNA polymerase (0.5 U/µl, Fig. 5A), dNTP (0.8 mM, Fig. 5B), Mg2 + (3.00 mM, Fig. 5C), FIP/BIP (2.00 µM, Fig. 5D), F3/B3 (0.40 µM, Fig. 6A), betaine (1.00 mM, Fig. 6B), and HNB (0.8 mM, Fig. 6C). According to the optimal concentrations of each component in the LAMP reaction system, the optimal reaction temperature and time were 63 °C (Fig. 7B) and 60 min (Fig. 7A), respectively. The HNB colorimetric assay results (optimized reaction conditions) demonstrate successful visual differentiation between controls (Fig. 8). The negative control (ddH₂O) and susceptible control (wild type, WT) both display a consistent baseline coloration, while the resistant control (mutant type, MT) exhibits a distinct color change. This clear visual contrast confirms the assay's specificity in detecting the target mutation, as the color shift occurs only in the presence of the mutant allele.

## Discussion

The escalating global reliance on insecticides has precipitated a concerning surge in pyrethroid resistance among *Ae. albopictus* populations. This significantly compromises the efficacy of chemical-based vector control strategies and positions insecticide resistance as a critical barrier to effective mosquito management worldwide. Given this urgent public health challenge, systematic resistance surveillance becomes imperative for formulating evidence-based control programs, as emphasized by Enayati et al. [12]. Our comprehensive resistance monitoring across 12 Shanghai districts revealed widespread pyrethroid tolerance in *Ae. albopictus* populations, with resistance detected in most surveyed locations. These findings underscore the urgent need to implement resistance mitigation strategies and alternative vector control approaches within urban mosquito management frameworks. Furthermore, observed variations in mortality rates across different pyrethroids likely reflect differences in selective pressure, application history, or resistance mechanisms (e.g., metabolic detoxification)—a relationship requiring further investigation.

Significant breakthroughs in understanding *kdr* mechanisms involve mutations in the VGSC gene. In 2011, the F1534C mutation was first detected in wild *Ae. albopictus* populations in Singapore, marking the initial discovery of *kdr* in this species [9]. Subsequent studies have uncovered geographical differences in mutation profiles. For example, in Chinese populations, new alleles have been identified, such as 1534S (serine) in Haikou and 1534L (leucine) in Shenzhen and Guangzhou [13–16]. At the same time, the I1532T mutation has appeared in both Italian and Chinese populations. Dual mutations at the I1532 and F1534 loci are thought to be potential adaptive responses to long-term insecticide use [13]. Correlations between genotypes and phenotypes show that the 1534S allele is strongly associated with resistance to permethrin and deltamethrin [14, 15]. In 2023, researchers employed clustered regularly interspaced short palindromic repeats (CRISPR)/CRISPR-associated protein 9 (Cas9) technology to engineer an F1534S substitution in a susceptible strain of *Ae. albopictus*. This genetic modification conferred resistance to deltamethrin. Subsequently, the reversion of this mutation led to the restoration of susceptibility in hybrid populations, clearly demonstrating that the F1534S mutation directly mediates resistance to deltamethrin [17]. The 1534S allele has emerged as a potentially valuable molecular marker for monitoring pyrethroid resistance in *Ae. albopictus* populations in China. Genetic characterization of VGSC domains II and III in Shanghai *Ae. albopictus* populations identified three *kdr*-associated missense mutations at codons 1016 (Val → Gly), 1532 (Ile → Thr), and 1534 (Phe → Ser/Cys), with 10 distinct genotypes observed: wild-type V1016V, heterozygous V1016G, and homozygous mutant G1016G at codon 1016; I1532I and I1532T at codon 1532; F1534F, F1534C, S1534S, and S1534C at codon 1534. The F1534S substitution dominated as the predominant mutant allele (allele frequency: 84.71%) in Shanghai. Data on combinatorial mutant genotypes and frequency distributions at the V1016, I1532, and F1534 loci in the *Ae. albopictus* VGSC gene reveal significant regional variations. For instance, genotype G5 (V/V + I/I + S/S) dominates in area BS (80.00% frequency), contrasting sharply with the distinct pattern observed in area CN. This disparity likely stems from local environmental factors, insecticide exposure, and genetic drift. Notably, with overall frequency of 52.55%, genotype G5 appears pivotal to *Ae. albopictus* biology and insecticide resistance mechanisms due to its impact on VGSCs. Future studies with larger sample sizes are warranted to strengthen data reliability, enabling a more comprehensive understanding of these genetic patterns and informing effective vector control strategies. These findings highlight the importance of combining traditional bioassays with molecular diagnostics. This integrated approach helps us understand the evolution of resistance and refine insecticide management strategies for controlling arboviral diseases.

While the V1016G *kdr* mutation in *Ae. albopictus* has been extensively validated as conferring significant resistance phenotypes (allelic effect size > twofold compared to F1534 variants, per Kasai et al. [10]), research focus on this locus remains limited in Chinese *Ae. albopictus* populations due to its low mutation frequency (< 5% in most surveys). In contrast, the F1534 codon has emerged as a critical resistance hotspot, with functional studies demonstrating pyrethroid resistance associations across three major alleles: F1534S and F1534L in Guangzhou populations [15], F1534C in Indian strains linked to DDT/deltamethrin cross-resistance [18], and analogous findings from Beijing and Haikou populations [19, 20]. This tripartite allelic convergence at F1534—spanning serine, leucine, and cysteine substitutions—reveals structural plasticity in domain III S6 helices that facilitates insecticide resistance through steric hindrance mechanisms, despite divergent regional selection pressures.

LAMP, a nucleic acid amplification technique for specific DNA sequences under isothermal conditions, was initially developed by Notomi et al. in 2000. This method employs specialized primers to generate loop structures and utilizes Bst DNA polymerase with strand-displacement activity at a constant temperature (~ 65 °C) [21, 22]. LAMP is characterized by its cost-effectiveness, procedural simplicity, rapid amplification, and visual interpretability, making it particularly promising for field-deployable diagnostic applications [23]. In recent years, LAMP-based assays have been successfully adapted for detecting insecticide resistance-associated mutations, including the acetylcholinesterase G119S mutation in *Nilaparvata lugens* (Stål) [24] and the GABA receptor R282S mutation in *Chilo suppressalis* [11], demonstrating its versatility in resistance monitoring. This study developed an AS-LAMP assay for rapid detection of the F1534S mutation in the VGSC gene of *Ae albopictus*. The LAMP reaction employed four primers (F3, B3, FIP, and BIP), with specificity validated using resistant (MT) and susceptible (WT) controls alongside ddH_2_O as a non-template control. LAMP analysis of 20 *Ae. albopictus* field samples utilized an HNB colorimetric indicator for genotype discrimination. Results demonstrated that 15 samples harboring the TCC/S-resistant allele exhibited a distinct blue color transition, while five wild-type homozygous samples retained the original purple coloration, consistent with the absence of amplification. The complete concordance between LAMP outcomes and Sanger sequencing validation confirmed the assay's accuracy in distinguishing resistant and susceptible genotypes.

Widespread pyrethroid resistance in Shanghai aligns with global trends. High F1534S frequency (84.71%) correlates with reduced insecticide binding affinity, while rare V1016G suggests emerging resistance. The LAMP assay offers a rapid, cost-effective alternative to sequencing for field monitoring. Future studies should explore metabolic resistance mechanisms and resistance management strategies.

## Conclusions

This study systematically investigated pyrethroid resistance and associated VGSC gene mutations in *Ae. albopictus* populations across 12 districts of Shanghai. Adult mosquito bioassays revealed widespread resistance to four pyrethroids (lambda-cyhalothrin, beta-cypermethrin, permethrin, and deltamethrin), with mortality rates below 80% in most regions. Genetic analysis identified high frequencies of the F1534S mutation (84.71%) and moderate V1016G (21.96%) in the VGSC gene. A LAMP assay was optimized (63 °C for 60 min) to rapidly detect F1534S mutations, demonstrating 100% concordance with sequencing. These findings highlight the problem of escalating pyrethroid resistance in urban mosquito populations and provide a field-deployable tool for resistance monitoring, supporting targeted vector control strategies to mitigate dengue transmission risks.

## Data Availability

Data supporting the main conclusions of this study are included in the manuscript.
